# Supplementary figures and images for: Soluble RAGE enhances muscle regeneration after cryoinjury in aged and diseased mice
Source: PLoS One. 2025 Feb 25;20(2):e0318754. doi: 10.1371/journal.pone.0318754 (PMC11856280; doi:10.1371/journal.pone.0318754)

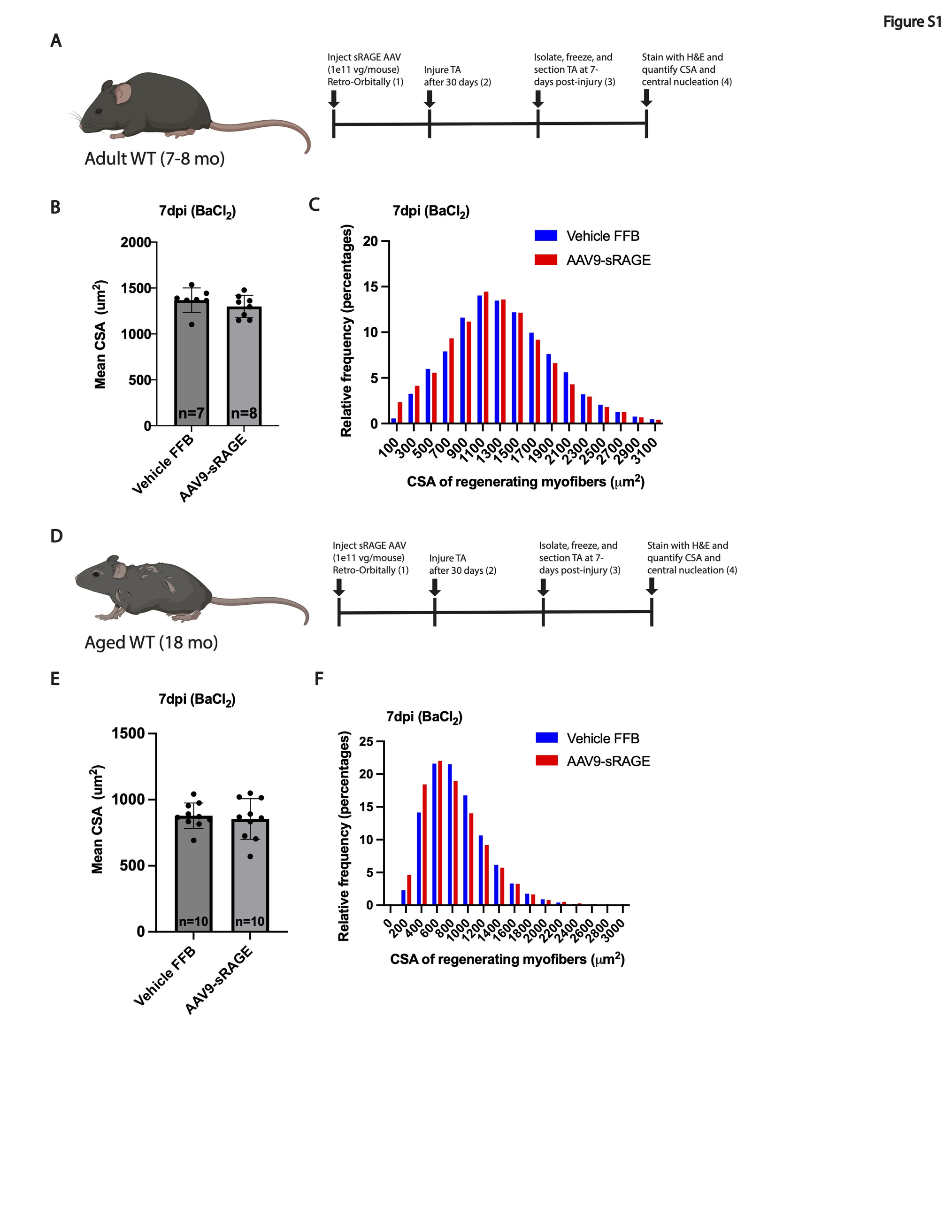

Supplement: S1 Fig — A) Experimental scheme for muscle regeneration assay in vehicle (FBB) and AAV9-sRAGE injected adult (7-8 months old) mice. B & C) Enumeration of the mean CSA (B) and distribution (C) of regenerating (centrally nucleated) muscle fibers in vehicle (FFB) and sRAGE treated, adult (7-8 months old) mice at 7 days after BaCl2 induced injury (n ≥ 7 male mice per condition). D) Experimental scheme for muscle regeneration assay in vehicle (FBB) and AAV9-sRAGE injected aged (18 months old) mice. E & F) Enumeration of the mean CSA (E) and distribution (F) of regenerating (centrally nucleated) muscle fibers in vehicle (FFB) and sRAGE treated, aged (18 months old) mice at 7 days after Bacl2 induced injury (n = 10 male mice per condition). Dots represent data for individual animals overlaid with mean ± SD. Data analyzed for statistical significance by Student’s two-tailed unpaired t test (B, E). Myofiber size distributions analyzed by Mann-Whitney U test (C, F). (TIF) [file pone.0318754.s001.tif]

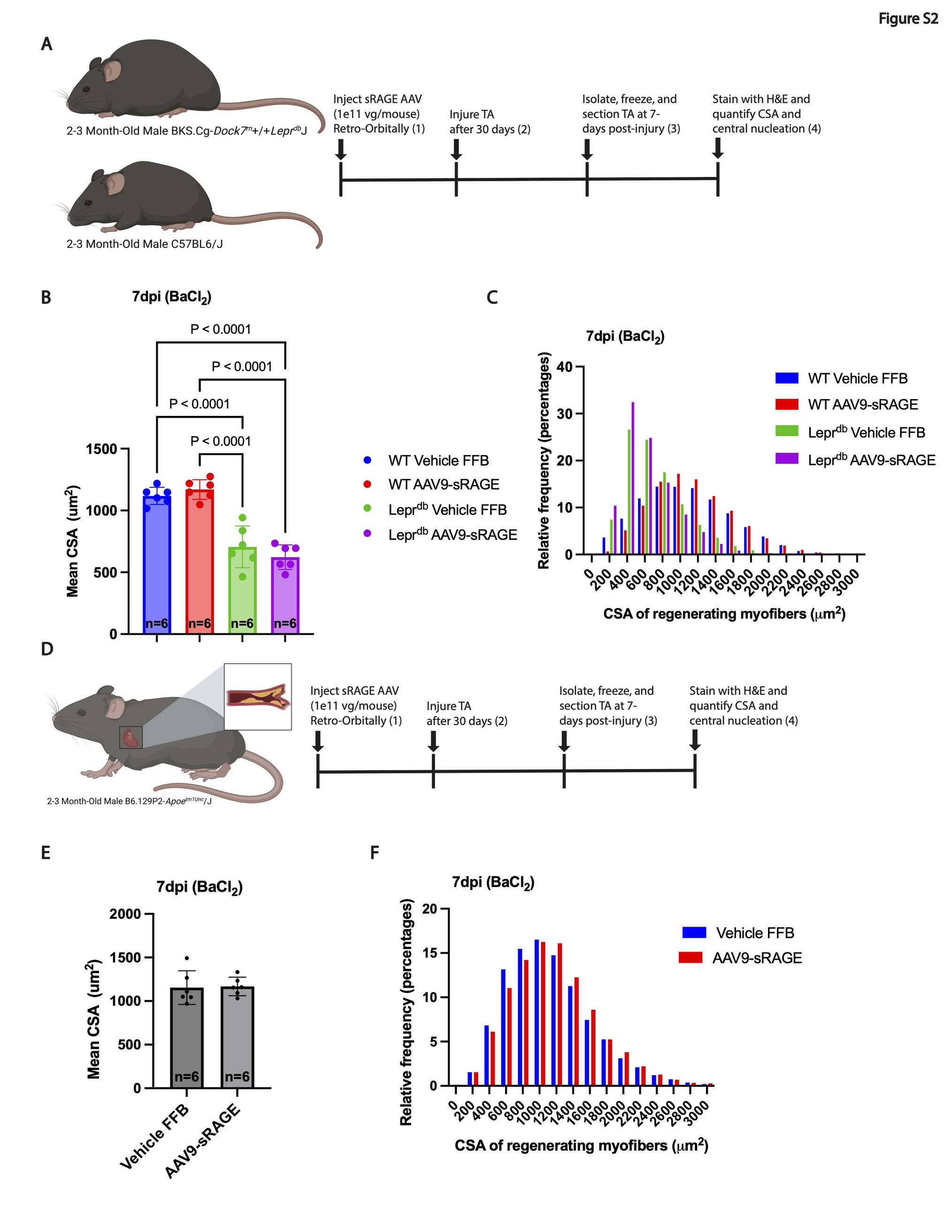

Supplement: S2 Fig — A) Experimental scheme for muscle regeneration assay in vehicle (FBB) and AAV9-sRAGE injected diabetic (Leprdb) and wild-type (WT) mice. B & C) Enumeration of the mean CSA (B) and distribution (C) of regenerating (centrally nucleated) muscle fibers in vehicle (FFB) and AAV9-sRAGE treated, diabetic (Leprdb) and wild-type (WT) mice at 7 days after BaCl2-induced injury (N = 6 male mice per condition). D) Experimental scheme for muscle regeneration assay in vehicle (FBB) and AAV9-sRAGE injected atherosclerotic (ApoE-null) and wild-type (WT) mice. E & F) Enumeration of the mean CSA (E) and distribution (F) of regenerating (centrally nucleated) muscle fibers in vehicle (FFB) and AAV9-sRAGE treated, atherosclerotic (ApoE-null) mice at 7 days after BaCl2-induced injury (n = 6 mice per condition). Dots represent data for individual animals overlaid with mean ± SD. Data analyzed for statistical significance by one-way ANOVA with Tukey post hoc test (B) and Student’s two-tailed unpaired t test (E). Myofiber size distributions analyzed by Mann-Whitney U test and Kruskal-Wallis test (C, F). All mice were 2-3 months of age at the time of study entry. (TIF) [file pone.0318754.s002.tif]

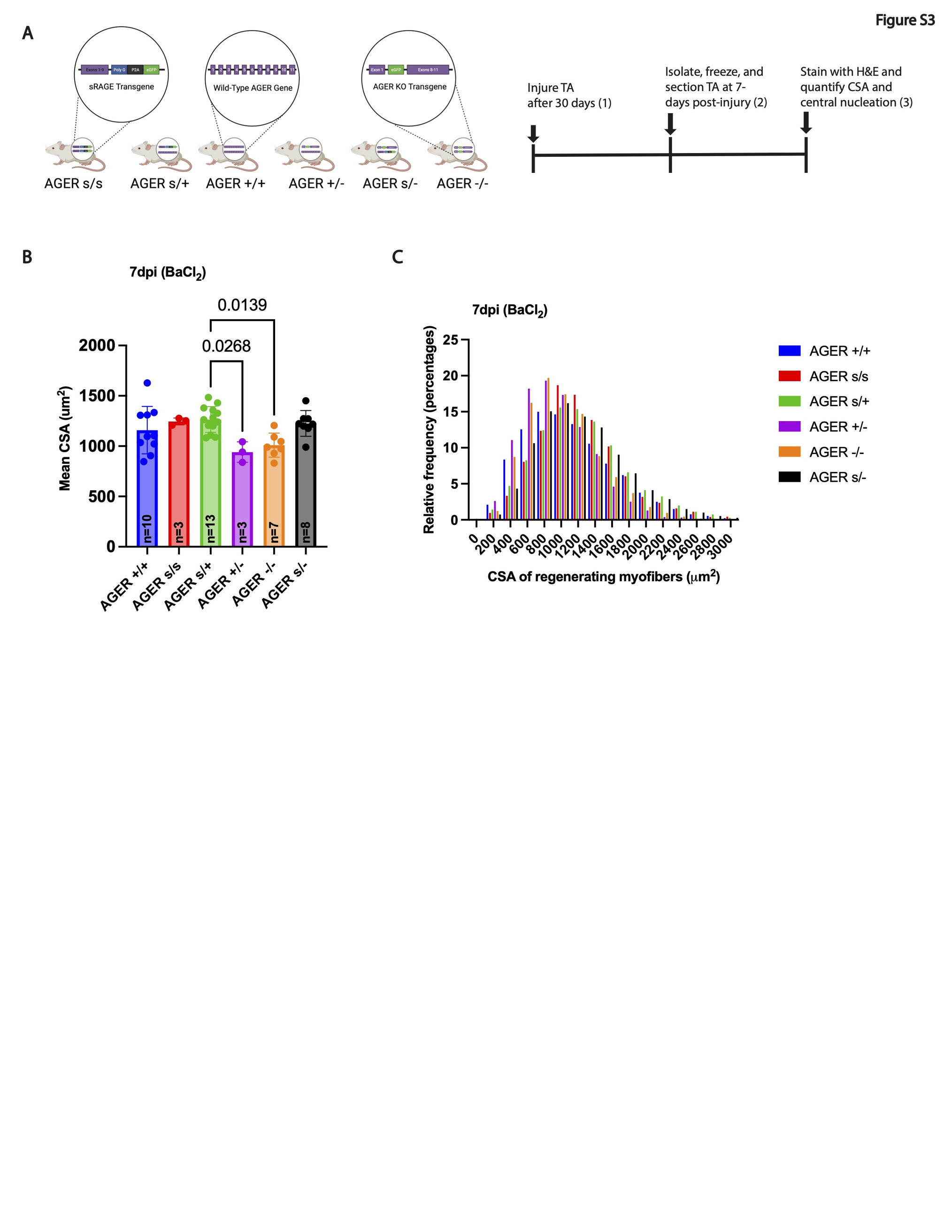

Supplement: S3 Fig — A) Experimental scheme for muscle regeneration assay in RAGE knockout and transgenic mice. B & C) Enumeration of the mean CSA (B) and distribution (C) of regenerating (centrally nucleated) muscle fibers in wild-type, knockout and transgenic mice at 7 days after Bacl2-induced injury (n ≥ 3 male mice per condition). Dots represent data for individual animals overlaid with mean ± SD. Data analyzed for statistical significance by one-way ANOVA with Tukey post hoc test (B). Myofiber size distributions analyzed by Kruskal-Wallis test (C). All mice were 7-8 months of age at the time of study entry. (TIF) [file pone.0318754.s003.tif]

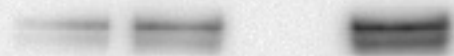

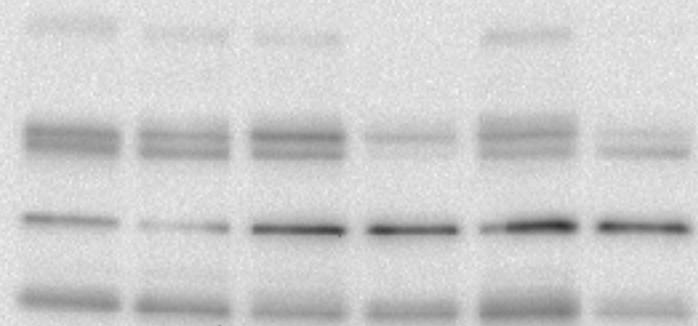

Supplement: S1 Raw images — (PDF) [file pone.0318754.s004.pdf]
